# Biological Effects of Tricalcium Silicate Nanoparticle-Containing Cement on Stem Cells from Human Exfoliated Deciduous Teeth

**DOI:** 10.3390/nano10071373

**Published:** 2020-07-14

**Authors:** Yoonsun Jung, Ji-Young Yoon, Kapil Dev Patel, Lan Ma, Hae-Hyoung Lee, Jongbin Kim, Jung-Hwan Lee, Jisun Shin

**Affiliations:** 1 Department of Pediatric Dentistry, College of Dentistry, Dankook University, 119 Dandae-ro, Cheonan, Chungcheongnam-do 31116, Korea; iamyoonsun93@gmail.com; 2 Institute of Tissue Regeneration Engineering (ITREN), Dankook University, 119 Dandae-ro, Cheonan, Chungcheongnam-do 31116, Korea; 72180459@dankook.ac.kr (J.-Y.Y.); kapildpatel20@gmail.com (K.D.P.); haelee@dku.edu (H.-H.L.); 3 Department of Nanobiomedical Science & BK21 PLUS NBM Global Research Center for Regenerative Medicine, Dankook University, 119 Dandae-ro, Cheonan, Chungcheongnam-do 31116, Korea; 4 UCL Eastman-Korea Dental Medicine Innovation Centre, Dankook University, 119 Dandae-ro, Cheonan, Chungcheongnam-do 31116, Korea; 5 Sounth China Center of Craniofacial Stem Cell Research, Sun Yat-sen University, Guangzhou 510055, China; malan6@mail.sysu.edu.cn; 6 Department of Biomaterials Science, College of Dentistry, Dankook University, 119 Dandae-ro, Cheonan, Chungcheongnam-do 31116, Korea

**Keywords:** calcium silicate-based cement, nanoparticles, stem cells from human exfoliated deciduous teeth, cytotoxicity test, odontogenic differentiation, dental pulp capping, deciduous teeth

## Abstract

Nanomaterials can enhance interactions with stem cells for tissue regeneration. This study aimed to investigate the biological effects of tricalcium silicate nanoparticle-containing cement (Biodentine™) during or after setting on stem cells from human exfoliated deciduous teeth (SHED) to mimic clinically relevant situations in which materials are adapted. Specimens were divided into four groups depending on the start of extraction time (during (3, 6 and 12 min) or after setting (24 h)) and extracted in culture medium for 24 h for further physicochemical and biological analysis. After cell viability in serially diluted extracts was evaluated, odontogenic differentiation on SHED was evaluated by ARS staining using nontoxic conditions. A physicochemical analysis of extracts or specimens indicated different Ca ion content, pH, and surface chemistry among groups, supporting the possibility of different biological functionalities depending on the extraction starting conditions. Compared to the ‘after setting’ group, all ‘during setting’ groups showed cytotoxicity on SHED. The during setting groups induced more odontogenic differentiation at the nontoxic concentrations compared to the control. Thus, under clinically simulated extract conditions at nontoxic concentrations, Biodentine™ seemed to be a promising odontoblast differentiating biomaterial that is helpful for dental tissue regeneration. In addition, to simulate clinical situations when nanoparticle-containing cement is adjusted, biological effects during setting need to be considered.

## 1. Introduction

Nanoparticle-based biomaterials have been applied in various biomedical fields, such as drug delivery, imaging, wound healing, tissue engineering, and dentistry, due to their improved physicochemical and mechanical properties and biological functionalities [1,2,3,4,5,6,7,8]. Especially in tissue regeneration, nanoparticle-based biomaterials can mimic nanoscale features of the natural extracellular matrix (ECM) and improve cell–material interactions with regenerating cells to form new tissues [9,10,11]. Moreover, nanoscale features can be used for targeted therapeutic delivery and imaging applications in dental and bone regeneration.

Premature loss of primary teeth may compromise the eruption of succedaneous teeth and lead to esthetic, phonetic, and functional problems. Although endodontically treated primary teeth may not cause severe problems, maintaining pulp vitality is desirable for several reasons [12]. When pulp vitality is maintained, it is possible to complete root development, initiate dentinogenesis to protect the pulp against various stimuli, and maintain a proprioceptive response [13]. Thus, in the case of primary teeth diagnosed with reversible pulpitis due to caries or trauma, vital pulp therapy can resolve the inflammation of the pulp, and pulp can return to a normal state [14]. Vital pulp therapies include indirect pulp capping, direct pulp capping, and pulpotomy with pulp regenerative materials. Pulp regenerative materials, called pulp capping materials, were reported to have regenerative effects on the dentine-pulp complex, which may be related to their ability to provide space for healing. Thus, they should have (i) great biocompatibility, (ii) calcium ion releasing ability for induction of mineralization, and (iii) other abilities (to induce pulpal cell injury for initiate immune and stem cell responses and suppress the growth of bacteria) [15].

Calcium hydroxide has been widely used with clinical success for pulp regenerative material since the 1930s [16]. Its ability to release hydroxyl (OH^−^) ions can cause local necrosis of the inflamed pulp tissue and inhibit microbial activity [17]. However, calcium hydroxide has been reported to form a dentin bridge along the pulp-material interface with multiple porosities and dissolve after long-term placement. These disadvantages may result in the failure of long-term biological seals against bacterial infection [18,19].

Mineral trioxide aggregate (MTA) was introduced and has been proven to have superior mechanical properties and the ability to induce more complete reparative dentin formation than calcium hydroxide [20]. However, several drawbacks of MTA as a pulp regenerative material include its long setting time (~240 min), poor handling, and coronal tooth discoloration [21]. A tricalcium silicate nanoparticle-containing cement, Biodentine™ (Septodont, Saint Maur des Fosses, France), has recently been introduced and publicized to overcome the drawbacks of the existing MTAs. In a previous study, it was reported that the size of particles in Biodentine was approximately 1–10 μm, but nano-sized particles were detected from a preliminary study using a particle size analyzer. These different results were caused by different resolution of particle sizes between backscattered electron (BSE) images and a particle size analyzer [22].

Biodentine™’s powder consists of tricalcium silicate, dicalcium silicate, calcium carbonate, and oxide filler such as iron oxide, and zirconium oxide [23]. Tricalcium silicate and dicalcium silicate are core material components, whereas zirconia oxide serves as a radiopacifier. Moreover, the setting time of Biodentine™ is shortened to 12 min after the addition of calcium chloride as an accelerator and polycarboxylate as a water-reducing agent [24]. Additionally, the incorporation of nanoparticles can increase the surface area and ionization, which is helpful for shortening the setting time. Polycarboxylate also increases the flow of the material and makes it easy for clinicians to handle the mixture by reducing the amount of water required for mixing [19]. Due to a substitution of zirconium oxide for bismuth oxide (which showed cytotoxic effects) as a radiopaque material, tricalcium silicate nanoparticle-containing cement (Biodentine™) exhibited less coronal discoloration than MTA [25].

Human pulp stem cells can be readily harvested from dental pulp tissue of extracted permanent teeth, which results in dental pulp stem cells (DPSCs), and exfoliated deciduous teeth, which are called stem cells from human exfoliated deciduous teeth (SHED), respectively [26,27]. DPSCs and SHED have attracted attention because they are ideal sources for dental tissue regeneration with the capacity of self-renewal and multilineage differentiation [28]. When the pulp is exposed by caries or trauma, pulp regenerative material can be applied on the exposed pulp and in contact with dental pulp connective tissue [29]. Occasionally, dental pulp cells can be damaged before, during or after dental procedures by, for example, bacterial infection (dental caries or trauma), iatrogenic injury (heat or mechanical force) or cytotoxic components (pulp regenerative materials applied above pulp tissue for vital pulp therapies) [30]. Thus, biocompatibility and bioactivity for pulp tissue regeneration have been carefully investigated during the development and usage of pulp regenerative materials [31,32]. The biocompatibility and bioactivity of Biodentine™ have been widely reported, with a focus on DPSCs [33,34]; however, only a few studies have focused on SHED [35]. The pulp tissues of both primary and permanent teeth are embryologically derived from the ectomesenchyme of the neural crest, but distinct differences between them have been reported [36]. SHED have been reported to be distinct from DPSCs by exhibiting higher proliferation ability and abundances of ECM and growth factors [37]. In addition, odontoblasts differentiated from SHED have been reported to show lower reparative activity than odontoblasts differentiated from DPSCs. It is assumed that SHED are likely more vulnerable than DPSCs [38].

Several studies have reported that tricalcium silicate nanoparticle-containing cement (Biodentine™) showed no cytotoxic effect as an extract in contact with DPSCs and SHED [35,39,40]. However, previous studies have not taken into account biological effects during setting because this material is usually applied on pulp cells from 3 to 6 min after mixing in cases of vital pulp therapies in clinics. This study aimed to investigate the biological effects of Biodentine™ during or after hydration in clinical circumstances using extracts obtained with different extraction starting times (3, 6, and 12 min and 24 h) after the start of mixing, focusing on cytotoxicity and differentiation on SHED and the causative factors from physicochemical analysis.

## 2. Materials and Methods

### 2.1. Preparation of Biodentine™ Specimens and Their Extracts

Biodentine™ capsules (Septodont, Saint Maur des Fosses, France) were mixed for 30 s according to the manufacturer’s instructions (Table 1). The mixtures were applied to Teflon molds, which were 2 mm in height and 10 mm in diameter and covered with overhead projector (OHP) film. Furthermore, the mixtures were divided into 4 groups with respect to the period from start of mixing to the start of extraction: during (3, 6 and 12 min) or after setting (24 h) (Table 2). After each setting period, Biodentine™ specimens were immediately placed into α-minimum essential medium (α-MEM; Gibco BRL, Grand Island, NY, USA) supplemented with 1% penicillin/streptomycin (Invitrogen, Carlsbad, CA, USA) for extraction. All Teflon molds and OHP films were sterilized with ultraviolet light for 1 h after ethylene oxide gas sterilization.

Each specimen was extracted with a extracted surface-area-to-extraction solution volume ratio of 3 cm^2^/mL as recommended in ISO 10993-12 for physicochemical and biological tests [41]. Because the surface area of the specimen was 2.2 cm^2^, it was incubated in 0.733 mL of culture medium. To mimic the clinical environment, all of the extracts were incubated for 24 h at 37 °C, 5% CO_2_, and 100% humidity. After 24 h, the supernatants were collected and filtered using a 0.2 μm Minisart^®^ syringe filter (Sartorius AG, Goettinggen, Germany) to exclude any extracted particles for further analyses. Before performing serial dilution, the medium was supplemented with 15% fetal bovine serum (FBS; Gibco, Waltham, MA, USA), 2 mM GlutaMAX (Gibco), and 1 mM L-ascorbic acid (Sigma, St. Louis, MO, USA). Additional penicillin/streptomycin was added to obtain 1% penicillin/streptomycin. The medium was subjected to serial dilutions to obtain the following extract concentrations for cytotoxicity tests: 50%, 25% and 12.5%. Biodentine™-free culture medium (α-MEM, Gibco) supplemented with FBS, penicillin/streptomycin, GlutaMAX, and L-ascorbic acid was incubated under the same conditions and served as the positive control group (0%). To prevent any possible contamination, all the experimental procedures were performed on a sterilized clean bench.

### 2.2. Physicochemical Analysis in a Cell-Free Culture Environment

#### 2.2.1. Powder Grain Size

To measure the particle size of the Biodentine™ powder, 3 capsules of powder were placed in distilled water (DW) using an electronic balance (Explorer EX224G, OHAUS, Parsippany, NJ, USA) and evenly dispersed in an ultrasonic cleaner (Power Sonic 410, Hwashin Technology, Seoul, Korea) for 30 min. Particle size measurements were performed using a particle size analyzer (LA-950V2, HORIBA, Kyoto, Japan). To collect the nanoparticles, Biodentine™ powder was placed in ethanol and centrifuged at 1000 rpm for 1 min. The supernatant was collected and dried for 24 h. The images of nanoparticles were taken by SEM (Sigma 500, ZEISS, Oberkochen, Germany) at magnifications of ×50,000. All analyses were performed with a 15 kV accelerating voltage.

#### 2.2.2. pH Measurements

Each Biodentine™ extract was collected after incubating for 24 h for pH measurements using a pH meter (inoLab pH 7110, WTW, Weilheim, Germany). The electrode was soaked into each Biodentine™ extract at room temperature (24 °C), and each measurement was repeated three times and averaged.

#### 2.2.3. Ion Release by ICP-AES

Ca, Si, Mg, Cu, Zn and P ions, which were detected in a previous study, were selected and their releases from each Biodentine™ extract were evaluated by measuring concentrations in medium with inductively coupled plasma-atomic emission spectrometry (ICP-AES; OPTIMA 8300, PerkinElmer, Boston, MA, USA) [35]. All the groups were analyzed three times, and the results were averaged.

### 2.3. Physical Analysis of Surfaces in a Cell-Free Culture Environment

#### 2.3.1. Scanning Electron Microscopy (SEM)

After extraction, Biodentine™ specimens were collected and dried for 24 h. Characteristics for each setting time were evaluated by SEM (Sigma 500, ZEISS, Germany). All analyses were performed with a 15 kV accelerating voltage. The images were taken at magnifications of ×100, ×500, ×5000 and ×10,000.

#### 2.3.2. Energy-Dispersive Spectroscopy (EDS)

The specimens that were collected for SEM were then analyzed by energy-dispersive spectroscopy (EDS; Noran System Seven, Thermo Fisher Scientific, Waltham, MA, USA) to detect the elements on the Biodentine™ surface. X-ray intensities were set at 100 counts per second, and the accelerating voltage was set at 15 kV.

#### 2.3.3. X-ray Diffraction (XRD)

A crystal structure assessment of the materials was performed using X-ray diffraction (XRD; Rigaku, Tokyo, Japan) with Cu Kα radiation at 40 mA and 45 kV from 20° to 40° of 2*θ* with a sampling width of 0.02° and scan speed of 0.5°/min. Phase identification was accomplished by consulting with search-match software utilizing the ICDD database (International Center for Diffraction Data, Newtown Square, PA, USA).

### 2.4. Primary Culture of SHED

Stem cells were isolated from vital primary teeth of healthy children (aged 5 years old, male, without any systemic disease and caries-free tooth) after written consent was obtained from their guardians. The primary mandibular incisor was extracted due to abnormal eruption of the permanent successor, which has been approved by the Ethical Committee off the Institutional Review Board of Dankook University Dental Hospital (IRB number DKUDH 2019-10-001). Since the primary incisor underwent physiological root resorption, the pulp was collected through the open subpulpal wall. For isolation of SHED from pulp, the enzymatic dissociation method was used, and the sorting by flow cytometry to purify of SHED was not performed, as previously described by Masako Miura et al. [42]. The minced pulp tissue was added to phosphate-buffered solution (PBS; Gibco, Grand Island, NY, USA) supplemented with 1% penicillin/streptomycin (Gibco). After incubation for 1 h at 37 °C with 2 mg/mL collagenase type I (Worthington Biochemical, Lakewood, NJ, USA) and 4 mg/mL dispase II (Invitrogen, Carlsbad, CA, USA), the solution was centrifuged at 1500 rpm for 3 min. The cells were cultured in α-MEM with 15% FBS, 1% penicillin/streptomycin, 2 mM GlutaMAX, and 1 mM L-ascorbic acid and incubated in a humidified atmosphere containing 5% CO_2_ at 37 °C. Cultured SHED in passages fewer than 10 passages were used for the experiments.

### 2.5. In Vitro Study of Biodentine™ on SHED

#### 2.5.1. Cytotoxicity by CCK-8 Assay and Staining by Phalloidin and 4′,6-Diamidine-2′-phenylindole Dihydrochloride (DAPI)

Cell cytotoxicity tests were performed according to ISO 10993-5 [43]. A total of 100 μL of 1 × 10^5^ cells/mL were cultured in each well of two 96-well plates (SPL Life Sciences, Pocheon, Gyeonggi-do, Korea) with supplemented medium in a humidified atmosphere of 5% CO_2_ at 37 °C for 24 h. After being washed with PBS (100 μL), the cells were cultured with 100 μL of serially diluted extract and positive control (0%) for another 24 h. The final percentages of extract in the culture medium were 50%, 25%, and, 12.5%, and supplemented medium was used as the positive control (0%).

Cell cytotoxicity was detected by the Cell Counting Kit-8 assay (CCK-8; Dojindo Laboratories, Kumamoto, Japan) [44,45]. Ten microliters of CCK-8 solution was added to each well, and the 96-well plates were placed in a CO_2_ incubator for 1 h to react. Then, the optical density (OD) for each well was determined at a wavelength of 450 nm to determine the cell viability on an iMARK™ Microplate Absorbance Reader (Bio-Rad, Hercules, CA, USA). The CCK-8 assay is based on measuring the dehydrogenase activity of living cells that are metabolically active and able to transform the slight yellow tetrazolium salt (WST-8) into orange-colored WST-8 formazan. All analyses were independently performed in triplicate, and representative images are shown. The cell viability was calculated as follows:

(1) $$Cell\;viability\left(\%\right)=\frac{OD\left(experiment\right)-OD\left(blank\right)}{OD\left(control\right)-OD\left(blank\right)}\times 100$$

After determining the cell viability by CCK-8 assay, the cells were washed with PBS, fixed with 4% paraformaldehyde (PFA), and permeabilized with 0.2% Triton X-100. Rhodamine phalloidin was applied and allowed to incubate for 25 min, and 4′,6-diamidine-2′-phenylindole dihydrochloride (DAPI) was added to the wells and incubated for 5 min. Cell images were obtained using an iRiS Digital Cell Imaging System (Logos Biosystems, Gyunggi-Do, Korea) at ×40.

#### 2.5.2. Odontogenic Differentiation and Biomineralization Evaluated by Alizarin Red S (ARS) Staining

SHED were seeded in 100 µL of growth medium onto a 96-well plate at a density of 1 × 10^4^ cells/600 µL. The cells used in this study were from the fourth passage. Furthermore, the culture medium was changed to odontogenic induction medium formulated using 12.5% diluted extract from Biodentine™ with different setting times (3, 6, 12 min and 24 h) the next day and every 3 days for 15 days. The odontogenic induction medium contained 0.1 µM dexamethasone (D4902, Sigma-Aldrich, St. Louis, MO, USA), 10 mM β-glycerophosphate (β-glycerophosphate disodium salt hydrate, G9422, Sigma-Aldrich), and 50 µg/mL ascorbic acid (L-ascorbic acid, A4544, Sigma-Aldrich).

At days 9 and 15 of odontogenic induction, biomineralization was evaluated using alizarin red S (ARS) staining. In brief, cells were fixed with 4% PFA for 10 min after PBS washing and rinsed with deionized water (diH_2_O) and then stained with 40 mM ARS (A5533, Sigma-Aldrich) solution (pH 4.1–4.3) at room temperature for 30 min. The ARS solution was removed, and the cells were washed with diH_2_O three times. The images were scanned using a scanner (EPSON Perfection V300 PHOTO, Suwa, Japan) and taken by light microscopy (Olympus lX71, Shinjuku, Tokyo, Japan). For the quantification of ARS staining, stained cells were destained in 10% *w*/*v* cetylpyridinium chloride (C0732, Sigma-Aldrich), and their absorbance was measured at 562 nm using a microplate reader. All analyses were independently performed in triplicate, and representative images are shown.

### 2.6. Statistical Analysis

Statistical analysis was performed using Kruskall-Wallis test and post hoc test of Mann-Whitney at *p* < 0.05. SPSS 23.0 (Statistical Package for Social Science, version 23.0, IBM Corporation, Chicago, IL, USA) was used.

## 3. Results and Discussion

### 3.1. Physicochemical Analysis in a Cell-Free Culture Environment

**Figure 1:**
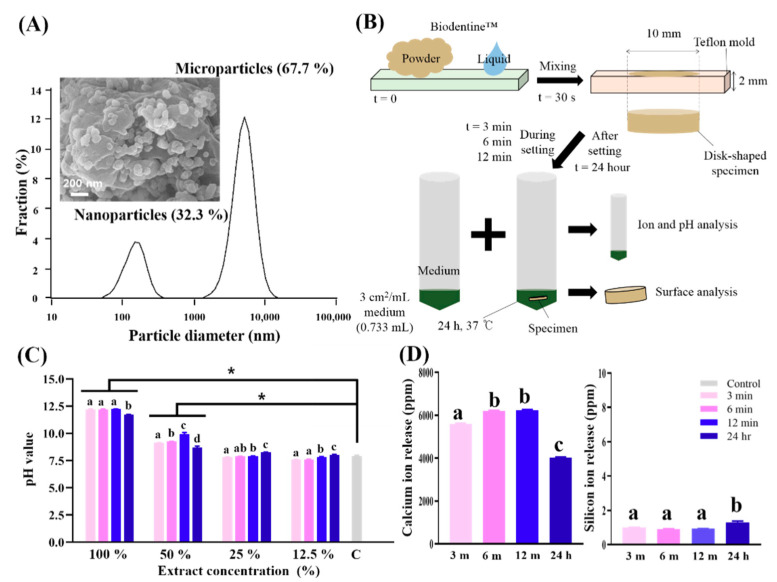
Schematic of this test procedure with different setting times for specimens and results of physicochemical analysis. (**A**) Particle size analysis of tricalcium silicate nanoparticle-containing cement (Biodentine™) powder confirmed that the powder was composed of nanoparticles and microparticles. Scanning electron micrograph (×50,000) of the Biodentine™ powder exhibited inhomogeneous microstructured surfaces with nanoparticles (approximately 100 nm). (**B**) The methodology used in the present study. To evaluate the biological effect of Biodentine™, the specimens were collected after 3, 6 and 12 min and 24 h from starting setting in the mold, immersed in medium and kept at 37 °C, 5% CO_2_ and 100% humidity. After 24 h, the extracts were collected and kept in 4 °C for use. (**C**) pH values of extracts with different dilution ratios. The asterisk indicates a significant difference between the control and experimental groups (*p* < 0.05). Biodentine™ increased the pH of medium to be highly alkaline, and the after setting group exhibited a lower pH value than during setting groups in the 100% and 50% extracts. (**D**) Ions released from the extracts as detected by inductively coupled plasma-atomic emission spectrometry. The concentrations of Ca and Si ions released in all of the extracts are shown, while Mg, Cu, Zn and P ions were not detected in any extracts. The numbers of Ca ions released increased during the setting and decreased after setting. The numbers of Si ions were negligible in the therapeutic range. Detected values (±SD) are analytically presented in tabular form underneath each bar. (**C**,**D**) Different letters indicate significant differences between groups (*n* = 3, *p* < 0.05).

The powder size was investigated to confirm that Biodentine™ contains nanoparticles. The powder particles had a mean size of 3770 ± 2500 nm, and a distinct second peak was observed at approximately 100 nm (Figure 1A). The SEM morphologies of the Biodentine™ powder exhibited inhomogeneous microstructured surfaces with nanoparticles (approximately 100 nm). The particle size measurement confirmed that the Biodentine™ powder was composed of nanoparticles (~32.3%) and microparticles (67.7%). These nanoparticles may penetrate through opened dentinal tubules to form the mineral infiltration zone and lead to increased mechanical properties of the interface and the permeation of several ions followed by increased mineralization [46].

To evaluate the biological effects of bioactive Biodentine™, the physicochemical properties of its extract were analyzed (Figure 1B). Bioactive Biodentine™ does not come in contact with so much moisture during setting in clinical situation but, according to ISO standard 10993-12 dealing with biomaterials’ extraction conditions (3 cm^2^/mL) [41], the Biodentin™ specimen was immersed in media, possibly mimicking or exaggerating moisture in clinical settings. The pH value of the extracts with different concentrations was measured initially. In medium with Biodentine™, the pH values were increased compared to those the control medium group which contains any extracts (7.91 ± 0.08) at 50 and 100% (*p* < 0.05) (Figure 1C). From the 100% extract, the pH value of the 24 h group (11.73 ± 0.02) was significantly lower than those of the 3 min (12.20 ± 0.03), 6 min (12.21 ± 0.03), and 12 min groups (12.23 ± 0.00, *p* < 0.05). A similarly low pH value in the 24 h group compared to during setting groups was also observed in the 50% extract (*p* < 0.05). These results confirm that Biodentine™ sets through a hydration reaction that produces hydrated calcium silicate gels and calcium hydroxide and then releases hydroxyl ions, causing an increase in pH [17,47]. This high alkalinity can produce an environment that is unfavorable for the survival and proliferation of bacteria [47]. Increases in pH and the release of various ions have been reported to induce the mechanism for reparative dentin formation by deposition of mineralized tissue [48]. Biodentine™ increased the pH of the medium to alkaline levels (~12) in a cell-free environment, which is similar to previously reported results [49].

Ca and Si ions are the main components of the Biodentine™ backbone and are released in all of the extracts; however, Mg, Cu, Zn, and P ions were not detected in any extracts (Figure 1D). The number of Ca ions released increased during the setting, but after setting (24 h), the number of Ca ions released significantly decreased (*p* < 0.05). Another study reported that Ca ions in a plain α-MEM medium were absorbed by Biodentine™ for 2 weeks [35]. Extracellular Ca ions in calcium hydroxide, unreacted calcium chloride, and other calcium containing silicates possibly modulate the gene expression of bone-related proteins during calcification in pulp [50,51]. Furthermore, the number of Si ions released increased after setting compared to the number in ‘during setting groups’, while the numbers of Si ions were negligible in the therapeutic range [52].

The pH value and number of released Ca ions exhibited similar patterns in this study. The numbers of Ca ions released and hydroxide ions (in accordance with the pH value) increased from 3 to 12 min (during setting); however, the numbers of Ca ions and hydroxide ions released decreased at 24 h (after setting). These ion release patterns could potentially be attributed to the setting reactions of Biodentine™. Hydration of Biodentine™ resulted in the formation of calcium hydroxide and calcium silicate hydrate gel [47]. When this hydration occurs at the surface of Biodentine™, unreacted tricalcium silicate grains are surrounded by layers of calcium silicate hydrated gel. Thus, unreacted tricalcium silicate grains are relatively impermeable to water, which thus slows down the effects of further reactions and decreases the numbers of Ca and hydroxide ions that can be released after setting. This pH and these ion releases need to be translated from the 100% extract to clinical circumstances because SHED might contact with Biodentine™ during pulp regenerative procedures. Due to the high alkalinity at 100% concentration, Biodentine™ might deleteriously affect surrounding pulpal cells or kill the underlying bacteria. From another point of view, when the concentration of Biodentine™ extract is diluted by body fluid below the therapeutic range, the bioactivity of Biodentine™ can be expected to be helped by the alkaline pH and Ca ions, promoting odontoblast-like cell differentiation and consequent biomineralization [53,54]. Moreover, the release of Ca ions from three different forms of tricalcium silicate (ProRoot^®^ white MTA, MedCem MTA^®^, and Biodentine™) have shown the dependency on exposed surface area (ESA), volume (V) and environmental pH [50]. The incorporation of nanoparticles increases the surface area and ionization which is beneficial for the setting. The released Ca ions from unreacted calcium chloride can shorten the setting time of Biodentine™.

### 3.2. Physical Analysis of Surfaces in a Cell-Free Culture Environment

**Figure 2:**
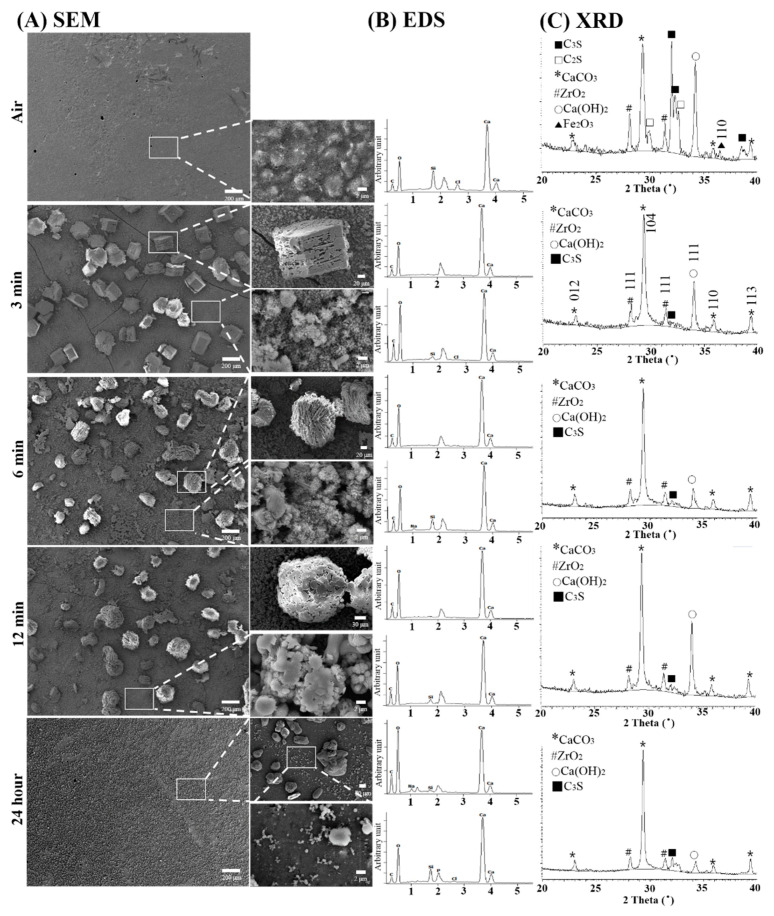
Physical analysis of tricalcium silicate nanoparticle-containing cement (Biodentine™) surface dried in the air for 24 h (Air) or immersed in medium for 24 h after setting for 3, 6 or 12 min or 24 h. (**A**) Scanning electron micrographs (×100, 500, 800, 10,000) of the Biodentine™ surface show a relatively uniform flat surface without microstructural particles for samples that had been in the air for 24 h, while the 3, 6 and 12 min and 24 h groups exhibited rough surfaces with various particles of different sizes in the micrometer range. (**B**) Energy-dispersive spectroscopy analysis revealed that calcium, oxygen and carbon were present in the scattered particles, but surface silicon was also present in the matrix. There was no significant difference in the exhibited elements according to the setting time (3, 6 and 12 min and 24 h groups). (**C**) X-ray diffractograms of Biodentine™ showed that the different phases tricalcium silicate, dicalcium silicate, calcium carbonate, zirconium oxide and calcium hydroxide are components of the 24 h air-dried Biodentine™ specimens; however, the 3, 6 and 12 min and 24 h groups exhibit mainly calcium carbonate, zirconium oxide and calcium hydroxide components, and there is a very small peak of tricalcium silicate for the 3, 6, 12 min and 24 h group. For Biodentine™, a close up of the 2*θ* region from 20° to 40° with a sampling width of 0.02° and a scan speed of 0.5°/min is shown embedded in the image.

After setting in air or being immersed in medium for 24 h, SEM, EDS and XRD were carried out to evaluate the physical properties of the Biodentine™ surface. The specimens set in air for 24 h exhibited a relatively uniform flat surface without microstructural particles, while the 3, 6 and 12 min and 24 h groups exhibited rough surfaces with various particles of different sizes in the micrometer range (Figure 2A). Moreover, the microparticle size decreased with increasing setting time and decreased in the 24 h group compared to the 3, 6 and 12 min groups. EDS analysis of the microstructural particles revealed the presence of calcium, oxygen, silicon and carbon (Figure 2B). The XRD results show that the phases tricalcium silicate, dicalcium silicate, calcium carbonate, zirconium oxide, and calcium hydroxide were components of the 24 h air-dried Biodentine™ specimens; however, the 3, 6 and 12 min and 24 h groups exhibited mainly calcium carbonate, zirconium oxide, and calcium hydroxide components and a very small amount of tricalcium silicate (Figure 2C). The SEM surface morphologies of the 3, 6, and 12 min groups exhibited inhomogeneous microstructured surfaces with different sized microparticles (180 ± 20 µm), which can be explained on the basis of XRD patterns (mainly the presence of microparticles of calcium carbonate and calcium hydroxide). However, the 24 h group showed approximately 10 times smaller microparticles (2 ± 0.2 µm), and the 24 h air-dried group did not show any microparticles. Mainly because of the long time, the reaction in the presence of oxygen and carbon dioxide can decompose tricalcium silicate and dicalcium silicate into calcium hydroxide and calcium carbonate, as confirmed by XRD patterns [50,55,56]. In the 24 h group, a relatively even surface exhibited significantly smaller grains compared to those of the during setting groups. Furthermore, the EDS analysis showed that calcium, oxygen and carbon were in the scattered particles, but they were also exhibited in the matrix of surface silicon. There was no significant difference in the exhibited elements according to the setting time (3, 6 and 12 min and 24 h groups). The XRD spectra exhibited peaks for calcium carbonate [57], zirconium oxide [58], and calcium hydroxide [47,59]. In the 24 h group, the peak for calcium hydroxide was relevantly lower than those in the during setting groups, and the surface was not deposited with microstructural particles, unlike the surfaces of the during setting groups. Thus, the scattered microstructural particles on the surface might be calcium carbonate or calcium hydroxide-containing crystals. In the 24 h group, a peak for tricalcium silicate was also exhibited. Previously, there were reports regarding the absence of dicalcium silicate phase in XRD analysis of Biodentine™ [47,60]. However, along with XRD analysis here, recent studies showed the presence of dicalcium silicate peaks from Biodentine™ [22,61].

### 3.3. In Vitro Study of Biodentine™ on SHED

**Figure 3:**
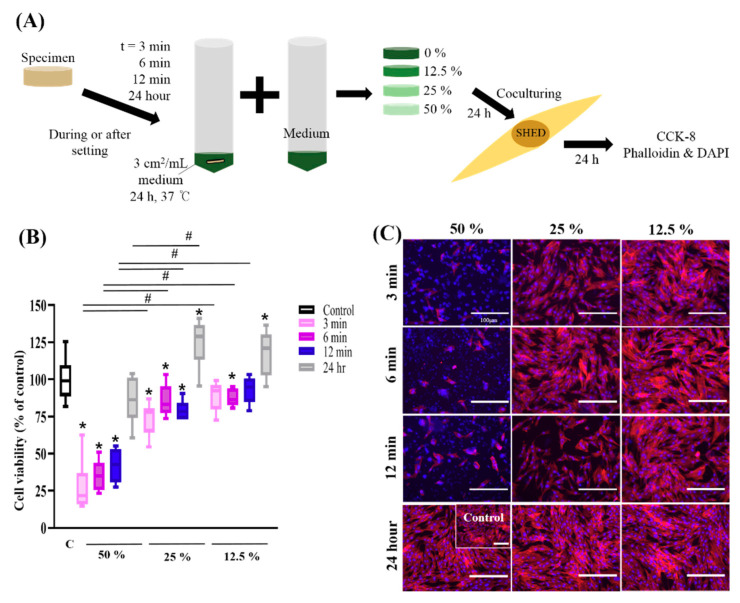
Schematic and results of initial (24 h) cytocompatibility of tricalcium silicate nanoparticle-containing cement (Biodentine™) with human exfoliated deciduous teeth (SHED). (**A**) To evaluate the cell viability of Biodentine™ at different dilution ratios (50%, 25% and 12.5%) and different setting times (3, 6, 12 min and 24 h), SHED were cultured with various groups for 24 h and then treated with Cell Counting Kit-8 assays and phalloidin and 4′,6-diamidine-2′-phenylindole dihydrochloride (DAPI) staining. (**B**) Cell viability of SHED. The 24 h group showed relatively higher cell viability than the 3, 6 and 12 min groups, and the control group (25% and 12.5%). Cell viability increased when the concentration of extracts decreased. The cytocompatibility during setting times shown in the 12.5% concentration group (3 and 12 min) was not significantly different from that in the control group. # indicates a significant difference between the same concentration groups, and the asterisk indicates a significant difference between the control and experimental groups (*p* < 0.05). (**C**) The results were reevaluated by confocal microscopy after staining with phalloidin and DAPI. Images of cells show that there are fewer live cells at 50% than in the control, confirming the cell viability assay. All of the 12.5% extracts showed similar cell viability to the control group (*n* = 3, *p* < 0.05). All scale bars represent 100 μm.

The initial (24 h) cytocompatibility of Biodentine™ was evaluated by measuring the cytotoxicity against SHED using the CCK-8 assay and was reevaluated by confocal microscopy images after staining with phalloidin and DAPI (Figure 3A). Figure 3B shows the cell viability results of the extracts of Biodentine™ without the 100% extract. Since Biodentine™ showed approximately 0% cell viability in 100% extracts, the results from the 100% extracts were excluded from the graphs and images. There was severe cytotoxicity for the 50% extract (27.6–41.9% cell viability), and cell viability increased when the concentration of extracts decreased. There was no significant difference in cell viability between the control and the 12.5% extract (3 and 12 min group) and the 50% extract (24 h group). Moreover, the cell viability for 25% and 12.5% extract (24 h group) was significantly higher than that for the control. To confirm the cell viability of the cultured conditions, confocal microscopy was used. The 24 h group showed relatively higher cell viability than the 3, 6 and 12 min groups. Cell images were taken after staining with DAPI and phalloidin to confirm the nucleus and cell morphology (Figure 3C). Images of cells showed fewer live cells at 50% in during setting group than in the control, confirming the cell viability assay. All of the images at 12.5% extracts showed similar cell viability to the control group.

It is generally assumed that cultured cells are susceptible to changes in pH and can survive at pH levels only between 6.6 and 7.8 [48]. Considering the high alkalinity shown at 100% and 50% in this study, the severe cytotoxicity shown at these concentrations is likely to be affected by high alkalinity. Many studies have reported that Biodentine™, which contacts cells after 24 h, showed no cytotoxicity on DPSCs [34] and SHED [35,62]. In this study, the results of the 24 h group are in line with the favorable cell viability on SHED reported in previous studies. However, to mimic the clinical situation, the setting times used in the present study were unlike those used in previous studies. The specimens were immersed in culture medium immediately after mixing for the experiments performed during setting (i.e., 3, 6 and 12 min). The present study showed cytotoxic effects of Biodentine™ on SHED at high concentrations in during setting group. Biodentine™ showed cytocompatibility (86.0–92.7% cell viability) for the during setting specimens in the 12.5% group. The results for the cell viability of serially diluted extract groups (50%, 25%, 12.5% and control) show that cytotoxicity is dose-dependent. This also made it possible to determine the most proper concentration considering the sensitivity of SHED. A total of 12.5% of Biodentine™ showed favorable results in this study.

**Figure 4:**
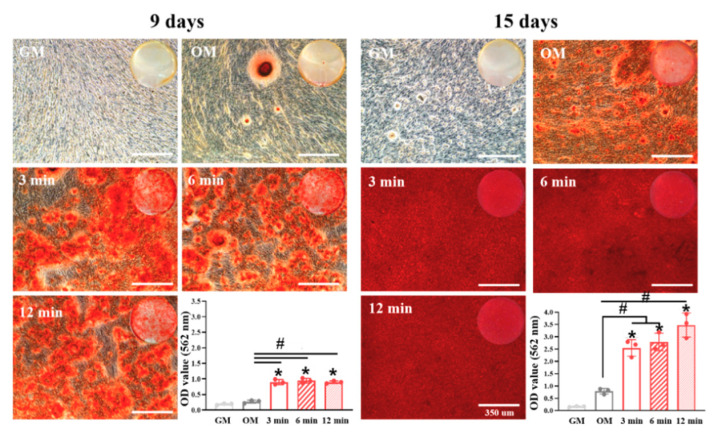
In vitro odontogenic differentiation by alizarin red S staining after 9 and 15 days of SHED exposure to tricalcium silicate nanoparticle-containing cement (Biodentine™) extracts. The mineral nodules formed remarkably in all the odontogenic media formulated with the 12.5% extraction group (3, 6 and 12 min) compared those in the growth medium, odontogenic medium, and after setting group at day 9. At day 15, the difference in mineralization in the odontogenic medium and extraction groups was observed more clearly. Optical images scanned by a photo scanner were added into the phase contrast images observed by microscope and quantification performed at 562 nm by a microplate reader. # indicates a significant difference between the odontogenic medium and experimental groups, and the asterisk indicates a significant difference between the growth medium and experimental groups (*p* < 0.05); All scale bars represent 350 μm.

The odontogenic differentiation of SHED was evaluated by ARS staining (Figure 4). Mineral nodules formed remarkably in all the odontogenic media formulated with the 12.5% extraction group (3, 6 and 12 min) compared those in the growth medium and even in the odontogenic medium at day 9. At day 15, the difference in mineralization in the odontogenic medium and extraction groups was observed more clearly. The OD 562 (optical density at 562 nm) values of all extraction groups were over 3 fold that of normal odontogenic medium (*p* < 0.05), and the value increased as the setting time increased, possibly due to there being more Ca ions in the extracts at 12 min than at other times. The extract from Biodentine™ thus revealed a significant increase in odontogenic potential under noncytotoxic conditions, revealing the promising roles of cement containing nanoparticles in the clinic. Other biological assays such as qRT-PCR and immunoblotting were not performed here because we successfully compared odontogenesis capacity of Biodentine™ extracts depending on setting conditions by ARS staining as a final marker of odontogenic differentiation. For future studies, in-depth biological study in terms of inflammation and differentiation ability is needed for supporting the safe use of nanoparticle-incorporated Biodentine™. Until now, there has been no case report related with immunogenicity concerns. However, the need for future studies regarding the immunogenicity of Biodentine™, especially during setting, will not be negligible after consideration of cytotoxicity.

## 4. Conclusions

In conclusion, extracts obtained from bioactive tricalcium silicate nanoparticle-containing cement (Biodentine™) during setting showed more cytotoxic effects on SHED than the extracts obtained after setting, while they induced more odontogenic differentiation at the nontoxic concentrations than the control group. The physicochemical properties analysis showed differences in the numbers of released Ca ions, pH changes, and surface chemistry among extraction conditions, supporting the abovementioned different biological functionalities. Thus, under clinically simulated extract conditions at nontoxic concentrations, Biodentine™ seemed to be a promising odontoblast differentiating biomaterial that is helpful for dental tissue regeneration. In addition, to simulate a dental clinical situation, the cytotoxicity and biological functions of biomaterials during setting need to be considered.

## Figures and Tables

**Table 1:** Composition and setting time of Biodentine™ as specified by the manufacturer.

Product Name	Composition			Setting Time	Mixing and Placement Time	Manufacturer
Biodentine™	Powder	Tri-calcium silicate	Main core material	12 min	6 min	Septodont
		Di-calcium silicate	Second core material			
		Calcium carbonate	Filler			
		Iron oxide	Shade			
		Zircornium oxide	Radiopacifier			
	Liquid	Calcium chloride	Accelerator			
		Hydrosoluble polymer	Water-reducing agent			

**Table 2:** Experimental conditions of Biodentine™.

Extraction Starting Time after Start of Mixing				Mixing Time	Mixing Speed	Setting Reaction
During Setting			After Setting			
3 min	6 min	12 min	24 h	30 s	4000 rotations/min	Hydration
